# Reasons for and consequences of missed appointments in general practice in the UK: questionnaire survey and prospective review of medical records

**DOI:** 10.1186/1471-2296-6-47

**Published:** 2005-11-07

**Authors:** Richard D Neal, Mahvash Hussain-Gambles, Victoria L Allgar, Debbie A Lawlor, Owen Dempsey

**Affiliations:** 1 North Wales Clinical School Department of General Practice, University of Cardiff, Wrexham Technology Park, Wrexham LL13 7YP, UK; 2 Clinical Trials Research Unit, University of Leeds, 17 Springfield Mount, Leeds LS2 9NG, UK; 3 Centre for Research in Primary Care, University of Leeds, 71–75 Clarendon Road, Leeds LS2 9PL, UK; 4 Department of Social Medicine, University of Bristol, Canynge Hall, Whiteladies Road, Bristol, UK; 5 Lockwood Research Practice, 3 Meltham Road, Lockwood, Huddersfield, West Yorkshire, HD1 3XH, UK

## Abstract

**Background:**

Missed appointments are a common occurrence in primary care in the UK, yet little is known about the reasons for them, or the consequences of missing an appointment. This paper aims to determine the reasons for missed appointments and whether patients who miss an appointment subsequently consult their general practitioner (GP). Secondary aims are to compare psychological morbidity, and the previous appointments with GPs between subjects and a comparison group.

**Methods:**

Postal questionnaire survey and prospective medical notes review of adult patients missing an appointment and the comparison group who attended appointments over a three week period in seven general practices in West Yorkshire.

**Results:**

Of the 386 who missed appointments 122 (32%) responded. Of the 386 in the comparison group 223 (58%) responded, resulting in 23 case-control matched pairs with complete data collection. Over 40% of individuals who missed an appointment and participated said that they forgot the appointment and a quarter said that they tried very hard to cancel the appointment or that it was at an inconvenient time. A fifth reported family commitments or being too ill to attend. Over 90% of the patients who missed an appointment subsequently consulted within three months and of these nearly 60% consulted for the stated problem that was going to be presented in the missed consultation. The odds of missing an appointment decreased with increasing age and were greater among those who had missed at least one appointment in the previous 12 months. However, estimates for comparisons between those who missed appointments and the comparison group were imprecise due to the low response rate.

**Conclusion:**

Patients who miss appointments tend to cite practice factors and their own forgetfulness as the main reasons for doing so, and most attend within three months of a missed appointment. This study highlights a number of implications for future research. More work needs to be done to engage people who miss appointments into research in a meaningful way.

## Background

Missed appointments are important for patients and staff, and have a prevalence of 4.5–6.5% of booked consultations in the UK [1-3]. Previous studies have identified a number of socio-demographic and health factors including mental illness, associated with missing an appointment, but have had important methodological problems such as lack of control groups, cross-sectional design and small numbers [4-8]. We have recently demonstrated how health professionals in the UK blame missed appointments on patients' mistakes and forgetfulness, and how patients who miss appointments are viewed negatively [9]. Only one study has investigated patients' issues related to missing appointments; participants in this study did not feel obliged to keep an appointment in part because they felt disrespected by the health care system, an effect which was compounded by participants' lack of understanding of the scheduling system [10]. No studies have reported explanations as to why socio-demographic variables should be associated with missed appointments, or data regarding the outcomes of missed appointments. Hence there is an insufficient evidence base to deal with missed appointments in primary care [11,12]. Furthermore, research from secondary care in the UK or primary care in the US is unlikely to be generalisable to UK primary care [4].

The aims of this study are to: (a) determine the reasons for missed appointments; (b) determine whether patients who miss an appointment subsequently consult their GP for the problem or symptom that was going to be presented in the missed appointment and (c) to compare psychological morbidity, and the previous number of missed appointments between individuals who miss an appointment and a comparison group who did not.

## Methods

### Main study design

The study was conducted over a three week period in 2001 in seven practices in West Yorkshire. These differed in terms of size, location, annual consulting rates, organisation of clinical workload, and missed appointment rate. Whilst all were members of a research network, it has recently been demonstrated that research practices do not differ from others in terms of their demography or morbidity [13].

All adult patients who missed an appointment and patients in the comparison group (the next adult seen in the same surgery session to control for: doctor-related factors, climate factors and practice factors) were sent a pack within 24 hours of the appointment, comprising a short and neutral invitation letter, and a questionnaire with open and closed questions about the missed appointment (cases only) and appointments in general. Patients with severe mental impairment, learning difficulties, severe emotional distress, or terminal illness were excluded. Two reminders were sent to initial non-responders.

A second mailing was sent to responders comprising a GHQ-12 [14], and a consent form to review medical records. Multi-centre and local ethical approval was received.

### Questionnaire

The first part of the questionnaire aimed to empower and engage the patients by asking their views on how practices could help patients keep appointments (data not reported in this paper). The second part (cases only) asked directly the reason for making the appointment, and the reasons for missing it. The third section (cases only) explored the reasons behind the missed appointment by asking their agreement with sixteen statements.

### GHQ-12 and data from medical records

Returned GHQ-12s were scored according to the manual [14]. Patients scoring three or more were sent a letter explaining that they may be suffering from mental health problems and were advised to seek help from their GP. For those providing written consent, data were abstracted from medical records three months after the index appointment, relating to whether they consulted for the stated reason of the missed appointment, the number of days from the missed appointment to the next consultation, and the number of missed and kept appointments in the twelve months prior to the index appointment.

### Sample size

The sample size calculation was determined by the difference in GHQ-12 scores between cases and the comparison group. In order to identify a high and low GHQ score difference of 20% between the groups at 5% significance level and 80% power, 200 pairs of patients and controls would be required. To allow for a likely response rate of approximately 60%, we estimated a sample size of 360 pairs.

### Data analysis

Free text data relating to reasons for missing appointments were read independently by three of the researchers (MH-G, OD and RDN) and the main themes discussed. It was apparent that for each patient, there was one main reason. A categorisation emerged that was applied across the data. This was then refined further and the final categories agreed. Comparisons between patients who missed appointments and their controls were assessed using conditional multiple logistic regression for matched pairs. Data relating to the numbers of missed and kept appointments for patients who were not registered for the entire year prior to their missed appointment were adjusted to allow for the proportion of the year that they were registered and rounded to the nearest integer.

## Results

### Reasons for missing an appointment

Of the 386 patients who missed appointments 122 (31.6%) returned a valid questionnaire. Table 1 shows the responses to statements provided in the questionnaire concerning factors contributing to the missed appointment. Over 40% said that they forgot the appointment and a quarter that they tried very hard to cancel the appointment or that it was at an inconvenient time. A fifth reported family commitments or being too ill to attend. Table 2 shows the categorisation of free text reasons for each of the 122 respondents. The largest categories were 'misunderstandings and mistakes', 'illness or personal circumstances', 'forgetting', and 'other commitments'. Of the misunderstandings and mistakes, the largest sub-category was 'by the practice', and included 'being unable to get through', 'had cancelled', 'did not have an appointment', and 'told wrong time or date'.

**Table 1:** Responses to statements about the specific missed appointment

Item:	N	n Yes	% (95% CI) answering Yes
I forgot about the appointment	89	44	49.4 (38.7, 60.3)
I tried very hard to cancel the appointment	81	24	29.6 (20.2, 40.8)
The appointment was at an inconvenient time	81	24	29.6 (20.0, 40.8)
I had family commitments	80	20	25.0 (16.0, 35.0)
I was too ill to attend	84	20	23.8 (15.2, 34.3)
The appointment wasn't with the doctor of my choice	76	12	15.8 (8.4, 2.6)
My GP asked me to come back on that day	78	11	14.1 (7.3, 2.4)
I was unable to get transport	78	11	14.1 (7.3, 2.4)
I overslept	79	11	13.9 (7.2, 2.4)
I did cancel before the appointment^a^	82	10	12.2 (6.0, 21.3)
My symptoms were better / problems resolved	79	9	11.4 (5.3, 20.5)
I was unable to get time off work	77	6	7.8 (2.9, 16.2)
I was there and did not miss the appointment^a^	80	5	6.3 (2.1, 14.0)
I was unable to get there because of the weather	78	2	2.6 (0.3, 8.9)
I was in hospital at the time	77	2	2.6 (0.3, 9.1)
I couldn't be bothered	76	1	1.3 (0.0, 7.1)

N: Number answering particular item; n Yes: number answering yes; CI: confidence interval

^a ^No evidence for this when medical records reviewed for those who provided consent.

**Table 2:** Analysis of free text comments about the specific missed appointment

	N
1. Misunderstanding and mistakes	36
By family member	2
By patient	14
By practice	20
	
2. Illness or personal circumstances	28
Self	17
Family	11
	
3. Forgot	26
No reason/excuse	17
Waited too long	2
Pre-occupied/distracted	7
	
4. Other commitments	9
	
5. Other	23
Overslept/late	6
Resolution of symptom	5
Not doctor of choice	2
Travel problems	2
No idea/don't know	3
Unclassifiable or blank	5

N: Number answering particular item; n Yes: number answering yes; CI: confidence interval ^a ^No evidence for this when medical records reviewed for those who provided consent.

### Consulting after a 'missed' appointment

Of the 122 patients who missed an appointment and responded, 57 (47%) consented to medical records review. Age and gender distributions between those who gave consent and those who did not were similar (both p values > 0.2). Of these, 52 (91.2%) patients subsequently consulted within three months of the index consultation. One third were in the first week and over half were within three weeks. 33 (63.5%, 95% CI 49.0% to 76.4%) consulted for the stated problem that was going to present in the missed consultation. Nineteen patients (36.5%, 95% CI 23.6% to 51.0%) had no record of consulting for the same reason, and it was unclear for five patients.

### Comparisons between cases and controls

The overall response rate among the comparison group (223, 58%) was greater than for cases, and there was a tendency for a greater proportion of the comparison group who responded, to also respond to the GHQ-12 (65.4% versus 50.8%), and to consent for medical record review (61.0% versus 45.1%). There were 27 case-control pairs who responded and provided data on the GHQ-12 and 23 pairs who responded and provided consent to medical records.

The odds of missing an appointment was lower for women compared to men (odds ratio (95% confidence interval) 0.67 (0.19, 2.36)), and the odds of missing an appointment decreased with increasing age (odds ratio (95% confidence interval) for an increase of 1 year in age 0.95 (0.91, 0.99)) though due to small numbers these differences were imprecise and the gender difference did not reach statistical significance at the conventional 5% level. The odds of missing an appointment were greater among those who had missed at least one appointment in the previous 12 months (5.88 (0.83, 41.36)), though again due to low response, this did not reach statistical significance at the conventional 5% level. This association was not substantively changed with adjustment for age, sex and the number of appointments made in the previous 12 months. There was a slight tendency for the odds of missing an appointment to be greater among those with a high GHQ-12 score (≥ 3), though because of small numbers the estimates were imprecise. With adjustment for age and gender the odds ratio (95% confidence interval) for missing an appointment comparing those with a high to a low GHQ-12 score was 1.14 (0.41, 3.15).

## Discussion

We found that the commonest reasons that patients give for missing appointments are mistakes and misunderstandings (frequently by the practice) and forgetfulness. The majority of patients who missed an appointment consulted their GP within three months, with most of these consulting for the original problem that they were originally going to present. The low number of matched case-control pairs led to the comparisons between those who missed appointments and those who did not having wide confidence intervals which included 1.0, and despite some large effect sizes, for example a 33% reduced odds for a woman compared to a man of missing an appointment, these data cannot exclude the role of chance. However, reporting these results is important since the low response rate has important implications for future work in this area and highlights the importance of developing ways of engaging the disengaged in primary care research.

### Study strengths and limitations

The age distribution of the comparison group was in keeping with national figures for those attending general practice, indicating that the practices participating in this study are representative of general practice as a whole [15]. One of the main strengths of this study is the investigation of an area which is important to primary care but which has been rarely investigated, perhaps because of anticipated difficulties. Further, we have attempted to improve upon previous studies with a prospective design, comparisons with a comparison group and use of a neutral place for correspondence. The main weakness of the study is the low response and the differential response between patients and the comparison group. A selection bias may exist between those who responded with respect to the outcomes of interest; this may be because of both social desirability bias and post-hoc rationalisations in their written responses. Responders may be over-represented by those whose missed appointment was a genuine practice mistake, and those who were less obliged to operate within the practices' booking systems being less likely to respond. Assuming that all 68% of non-responders did not miss their appointment because of forgetfulness or practice misunderstandings, then interventions aimed at dealing with these problems would only be able to reduce the overall missed appointment rate by a small amount.

### Reasons for missing appointments

Within the category 'misunderstandings and mistakes', the largest sub-category was 'by the practice'. Further, in the response to the pre-defined categories, 30% stated that they had tried to cancel their appointment. A small number of patients reported that they had either cancelled their appointment or indeed kept their appointment. These findings were not verified by the medical records, therefore it is difficult to know whether these represent practice mistakes of post-hoc rationalisations of behaviour. Whilst this may reflect post-hoc rationalisations or may be exaggerated by selection bias, there is consistency between the two questions and the results suggest that improvements in practice communication systems could reduce missed appointments. Forgetfulness was another important cause. One practice strategy that may reduce this problem would be the use of aide-memoirs. However, intervention studies are required to demonstrate their effect. In a parallel study we have assessed the perceptions of primary care health professionals of the reasons for missing appointments [9]. Interestingly, practice staff tended to blame patients for missed appointments and dismissed the idea that practice factors may contribute. The results of this study suggest that practice factors contribute to some missed appointments suggesting that considering practice level interventions (e.g. making it easier to cancel appointments, and greater convenience of appointment times) may be useful.

### Consequences of missed appointments

The finding that virtually all patients who missed an appointment did subsequently consult within a three month period has not been previously demonstrated. In fact, over half of those who responded consulted within the following two weeks of the missed appointment. Again selection bias may have influenced this result if those who did not respond were different to those who did and were less likely to subsequently attend. This study was unable to determine whether those with psychiatric morbidity are more likely to miss appointments; and whilst further research in this area is required to determine this, a recent paper has found that psychological morbidity did affect the likelihood of missing appointments [7].

### Engaging with the disengaged

There are several implications of these results for future research. This work adds to the literature confirming that more work needs to be done to engage people who miss appointments with research in a more meaningful way. Creative approaches, in terms of accessing and engaging patients who have missed appointments in research, and of using appropriate methodologies to answer important questions need developing; qualitative methods may be best at exploring the phenomenon from user perspectives and would help to unravel the complex relationships surrounding consultation etiquette and patients' and practitioners' behaviour. Closer involvement of users might inform this process [16], as may the use of inducements. One option would be to recruit patients when they next consult in primary care, as we have shown the vast majority do consult again soon. If research was then conducted in a neutral environment, the use of practice members to recruit participants need not bias the responses. The lessons that we have learned have implications for future research for others who are developing work with other 'disengaged' or potentially vulnerable groups of patients. There is still a need for health professionals to maintain their concern for the health of patients who miss appointments, since this study does not exclude the possibility that they may have unmet mental health problems.

## Conclusion

Patients who miss appointments tend to cite practice factors and their own forgetfulness as the main reasons for doing so, and most attend within three months of a missed appointment. This study highlights a number of implications for future research. More work needs to be done to engage people who miss appointments into research in a meaningful way.

## List of abbreviations used

GP – General practitioner; GHQ – General Health Questionnaire

## Competing interests

The author(s) declare that they have no competing interests.

## Authors' contributions

The study was designed by RDN, DAL, VLA and OD. MH-G conducted the fieldwork under the direction of RDN. Data analysis was conducted by DAL and VLA. RDN and MH-G drafted the paper, which has been seen and critically revised by all co-authors.

## Pre-publication history

The pre-publication history for this paper can be accessed here:


